# The Low Noise Limit in Gene Expression

**DOI:** 10.1371/journal.pone.0140969

**Published:** 2015-10-21

**Authors:** Roy D. Dar, Brandon S. Razooky, Leor S. Weinberger, Chris D. Cox, Michael L. Simpson

**Affiliations:** 1 Gladstone Institute of Virology and Immunology, San Francisco, California, United States of America; 2 Department of Bioengineering, University of Illinois at Urbana-Champaign, Urbana, Illinois, United States of America; 3 Institute for Genomic Biology, University of Illinois at Urbana-Champaign, Urbana, Illinois, United States of America; 4 Center for Nanophase Materials Sciences, Oak Ridge National Laboratory, Oak Ridge, Tennessee, United States of America; 5 Bredesen Center for Interdisciplinary Research and Graduate Education, University of Tennessee, Knoxville, Tennessee, United States of America; 6 Laboratory of Immune Cell Epigenetics and Signaling, The Rockefeller University, New York, New York, United States of America; 7 QB3: California Institute for Quantitative Biosciences, University of California San Francisco, San Francisco, California, United States of America; 8 Department of Biochemistry and Biophysics, University of California San Francisco, San Francisco, California, United States of America; 9 Department of Civil and Environmental Engineering, University of Tennessee, Knoxville, Tennessee, United States of America; 10 Department of Materials Science and Engineering, University of Tennessee, Knoxville, Tennessee, United States of America; University of California San Diego, UNITED STATES

## Abstract

Protein noise measurements are increasingly used to elucidate biophysical parameters. Unfortunately noise analyses are often at odds with directly measured parameters. Here we show that these inconsistencies arise from two problematic analytical choices: (i) the assumption that protein translation rate is invariant for different proteins of different abundances, which has inadvertently led to (ii) the assumption that a large constitutive extrinsic noise sets the low noise limit in gene expression. While growing evidence suggests that transcriptional bursting may set the low noise limit, variability in translational bursting has been largely ignored. We show that genome-wide systematic variation in translational efficiency can–and in the case of *E*. *coli* does–control the low noise limit in gene expression. Therefore constitutive extrinsic noise is small and only plays a role in the absence of a systematic variation in translational efficiency. These results show the existence of two distinct expression noise patterns: (1) a global noise floor uniformly imposed on all genes by expression bursting; and (2) high noise distributed to only a select group of genes.

## Introduction

**Fig 1:**
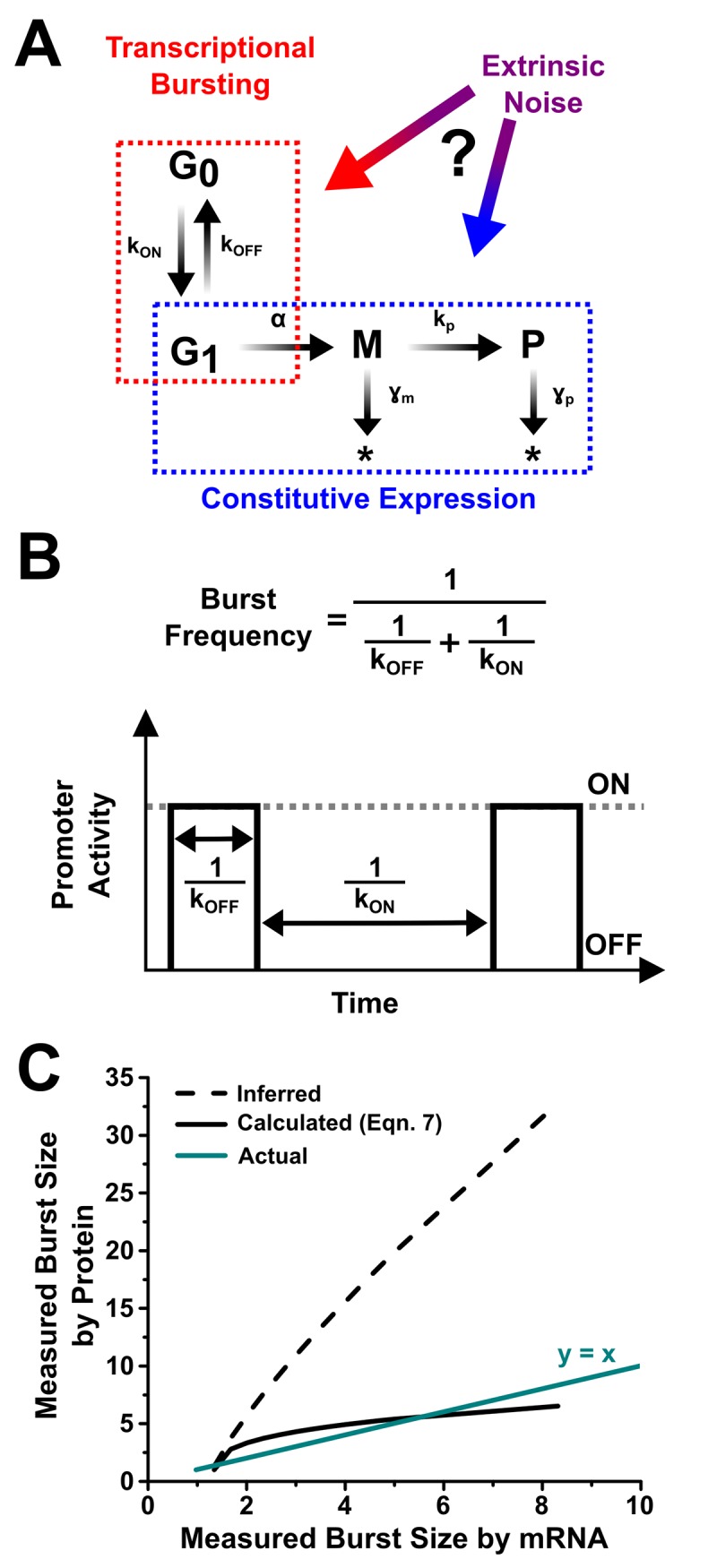
Assumptions of extrinsic noise coupling reveal a disparity in inferred versus actual transcriptional burst size measurements. **(A)** Transcriptional bursting (red dashed box) occurs when a promoter stochastically switches between an ‘OFF’, G_0_ state, and ‘ON’, G_1_ state, at rates *k*_*OFF*_ and *k*_*ON*_. In the G_1_ state mRNA, M, is transcribed at rate *α*, and translated into protein, P, at rate *k*_*p*_. mRNA and protein decay at rates γ_*m*_ and *γ*_*p*_ respectively. Constitutive expression (blue dashed box) is made of the processes of transcription from the G_1_ state, translation, and decay of M and P. Extrinsic noise, i.e. global fluctuations in shared resources, can potentially affect transcriptional bursting, constitutive expression, or both. **(B)** Schematic representation of promoter transitioning as a square wave where the average timing between bursts, T_OFF_, is 1/ *k*_*ON*_. The average duration of a burst, T_ON_, i.e. time in the ON, G_1_ state, is 1/ *k*_*OFF*_. The average number of bursts over a length of time is termed the transcriptional burst frequency. **(C)** Measured transcriptional burst size by protein versus mRNA measurements. The inferred trend (dashed line) shows the discrepancy from the true values (y = x, cyan line). Calculated values based on the corrected and reported model agrees well with the true values (solid line).

In principle the structure of noise in protein populations can be used to infer the architecture and dynamics of the underlying gene circuits and networks [1, 2]. However, inference is indirect, requires trust in analytical models, and may require reliance on assumptions. Despite the indirect approach, these analytical models have demonstrated some *qualitative* successes, but undoubtedly suffer from *quantitative* problems. A particularly relevant example from contemporary research is transcriptional bursting (Fig 1A); a model of transcription where multiple mRNAs are produced in episodic bursts separated by prolonged periods of inactivity (Fig 1B). Burst dynamics have been inferred using analytical models from reporter protein noise measured in bacteria [3, 4], yeast [3, 5], and mammalian cells [3, 6–8]. Although the main purpose of their analysis was burst frequency saturation, Sanchez and Golding demonstrated the large discrepancies between mRNA burst sizes inferred from protein noise measurements and from those measured more directly [3]. As a result of such inconsistencies, many researchers choose to disregard expression patterns extracted from protein noise measurements.

These more direct measures of transcriptional bursting are performed by characterizing mRNA production dynamics. In an elegant example, individual mRNAs were directly imaged with single-molecule resolution in living bacteria [9]. The live-cell mRNA method has been successfully adapted in yeast [10], social amoebae [11] and mammalian cells [12], allowing direct quantification of the number of mRNA produced during burst events. As implementation of a live-cell method can be difficult, noise in mRNA populations have also been measured using single-molecule fluorescence *in situ* hybridization [4, 13, 14]. Extensions of the single-molecule FISH approach are able to also calculate the ON and OFF time distributions of promoters through hybridization techniques [13]. Unfortunately, again there are inconsistencies between inferences of bursting dynamics from protein noise measurements and these mRNA measurements: e.g. there is an approximate factor of 4 transcriptional burst size difference between inferred and more directly measured transcriptional burst dynamics in *E*. *coli* [3, 4, 15] (Fig 1C).

One clear message is that the quantitative inference of transcriptional burst dynamics from protein noise measurements should be viewed with considerable skepticism. But perhaps the more important message is that the inability to infer transcriptional burst dynamics from protein noise data is a stark illustration of an incomplete analytical understanding of the connection between transcriptional bursting and the fluctuations in the associated protein populations. That is, if transcriptional burst dynamics cannot be accurately predicted from the noise in the protein population, it is difficult to then argue that the protein noise can be accurately predicted from the measured transcriptional burst dynamics. This can be an issue of great significance as transcriptional bursting may be the dominant (or at least an important) noise source, but the consequences of this noise may be realized in the protein population. For example in the HIV LTR promoter, although the noise of transcriptional bursting may set the noise behavior of this gene circuit, it is the noise in the HIV regulator Tat protein that interacts with the positive feedback within this circuit and may play a pivotal role in the establishment of proviral latency [16–18]. The understanding of this important gene circuit can only be complete when there is internal consistency in the analytical framework that connects transcriptional bursting and the protein noise behavior.

In addition to the caution needed when inferring transcriptional burst dynamics from protein noise measurements, theoretical analyses suggest that biophysical parameters cannot be inferred by static steady-state noise measurements (e.g. Flow cytometry, smFISH imaging, etc.) [19]. Yet, many experimental studies have used static protein or mRNA measurements to estimate transcriptional burst parameters [3, 4, 7, 15, 20]. Recently we have shown that noise magnitude quantified for clonal T-cell populations expressing a destabilized GFP using flow cytometry and time-lapse fluorescence microscopy are directly correlated (Supplementary Information of [6]). This observation suggests that at least in some experimental settings static and dynamic noise measurements, at least for cases of transcriptional bursting and constitutive gene expression, display some degree of ergodicity and enables inference of biophysical parameters from both static and dynamic measurements.

The analytical framework most often used in experimental studies to connect transcriptional bursting and protein population noise is the two-state (or random telegraph) model [21, 22]. This model has three transcriptional parameters described by rates of transition into (k_ON_) and out of (k_OFF_) activity, transcribed at rate α (Fig 1A). Assuming that k_OFF_ >> k_ON_ [6, 20] (S1 File)

$$CV_{i}^{2}=\frac{b_{i}+1}{<P_{i}>}\left(B_{i}\right)+E,$$ (1)

where B_i_ is the transcriptional burst size (average number of mRNA produced per transcriptional activity pulse), b_i_ is the translational burst size (average number of proteins produced per mRNA molecule), and *i* is used as an index associating each term with its respective gene. The first term on the right hand side of Eq 1 accounts for the noise associated with intrinsic constitutive expression and transcriptional and translational bursting (collectively referred to as burst noise). The E term represents noise that couples into the expression of all genes, even those that exhibit little (i.e. B_i_ ~ 1) transcriptional bursting. This E term should not be confused with the extrinsic noise measured using the two-reporter approach [23] as some portion of burst noise may be extrinsic as well (Fig 1A). To clearly differentiate the E term from the total extrinsic noise we will refer to it as the *constitutive* extrinsic noise (i.e. extrinsic noise *not* associated with the timing of expression bursting). This would include sources such as partitioning at cell division [24], variations in growth rate [25], mitochondria [26], and RNA polymerase concentration [27].

Eq 1 may be rearranged to solve for (i.e. infer) transcriptional burst sizes such that

$$B_{i}=\frac{<P_{i}>}{b_{i}+1}\left(CV_{i}^{2}-E\right).$$ (2)

In this approach, the noise magnitude ($CV_{i}^{2})$ and the protein population (< *P*_*i*_ >) are measured quantities. As noted above, theoretical analyses suggest that Eq 2 is more of a qualitative than a quantitative relationship [19, 28], and accordingly here we will not attempt to apply the relationship of Eq 2 to the detailed noise analysis of individual genes, but instead use it only to infer genome-wide patterns. Total extrinsic noise may be measured using the two-color method, but it is entirely unclear how much of this noise is constitutive extrinsic noise and how much of it is entangled in expression bursting. As a result, the selection of the E term has relied on one of two mutually exclusive assumptions. One group of investigators have apparently assumed E = 0. These investigators have focused mostly on measuring the noise from a limited number of promoters or in a reporter protein population [8, 14, 29–31]. Other investigators have focused on genome-wide noise measurements and have interpreted the data to indicate [4, 32] or have assumed [3] that E has a constant value large enough to dominate noise behavior for moderately and highly expressed proteins. Unfortunately these two assumptions lead to very different values for the inferred transcriptional burst sizes.

A second difficulty lies in the relationship between translational burst sizes and protein abundances. While much has been reported about the tendency for transcriptional burst sizes to increase as protein populations increase [6, 12, 15], similar studies linking translational burst sizes and protein abundances seem to be lacking. Certainly for many studies this lack of focus on translational bursting is simply a matter of experimental design. Studies that focus on mRNA populations or those that look at reporter protein populations are obviously not set-up to observe the relationship between translational burst sizes and protein abundances. Instead, only genome-wide measurements of mRNA and protein abundances and lifetimes can shed light on this important relationship which connects transcriptional bursting to fluctuations at the protein level. Where the relationship between translational bursting and protein abundance has been considered at all, it appears that investigators have assumed that there was no connection between these two parameters [3, 4, 33]. Indeed, this assumption is central both to the finding of a constitutive extrinsic noise floor in genome-wide noise measurements in *E*. *coli* [4] and in the inference of transcriptional burst sizes from measured protein noise [3].

Here we examine the relationship between translational burst sizes and protein abundances. We show that a genome-wide systematic variation in translational efficiency can–and in the case of *E*. *coli* does–play a significantly larger role than transcriptional burst size variation in controlling noise. Indeed, some of the inconsistencies in analytical models have been caused by misidentifying increased translational burst sizes as transcriptional burst size changes. Furthermore, the finding of a substantial constitutive extrinsic noise floor in *E*. *coli*–which was clearly feasible when this translational efficiency variation was not considered–can now clearly be ruled out. Instead we find that the noise floor in *E*. *coli* is indicative of transcriptional burst frequency saturation. In contrast, the noise in most yeast proteins continues to decline with increasing abundance down to a small extrinsic noise floor. High noise at high abundance in yeast is not a global feature, but instead is seen in a select group of proteins and is heavily dependent on promoter architecture [30, 34]. We show that this contrast with *E*. *coli* emerges first from the lack of a large systematic translational efficiency variation in yeast, and as suggested before [3] demonstrates that yeast shows no sign of burst frequency saturation. However, promoter-controlled noise does not preclude burst frequency saturation as we show data from human T lymphocytes that demonstrate roles for both frequency saturation and promoter architecture in controlling the noise lower limit. The picture that emerges from across these cell types is one where constitutive extrinsic effects (fluctuations in global resources that couple into constitutive gene expression processes) are uniformly small. Conversely, systematic translational efficiency variations or burst frequency saturation may have much larger effects. These findings remove the need for flawed assumptions that have impeded genome-wide application of the two-state model and provide an analytical framework that may be trusted to accurately infer genome-wide expression patterns from protein noise measurements.

## Results

### Global expression burst structure

**Fig 2:**
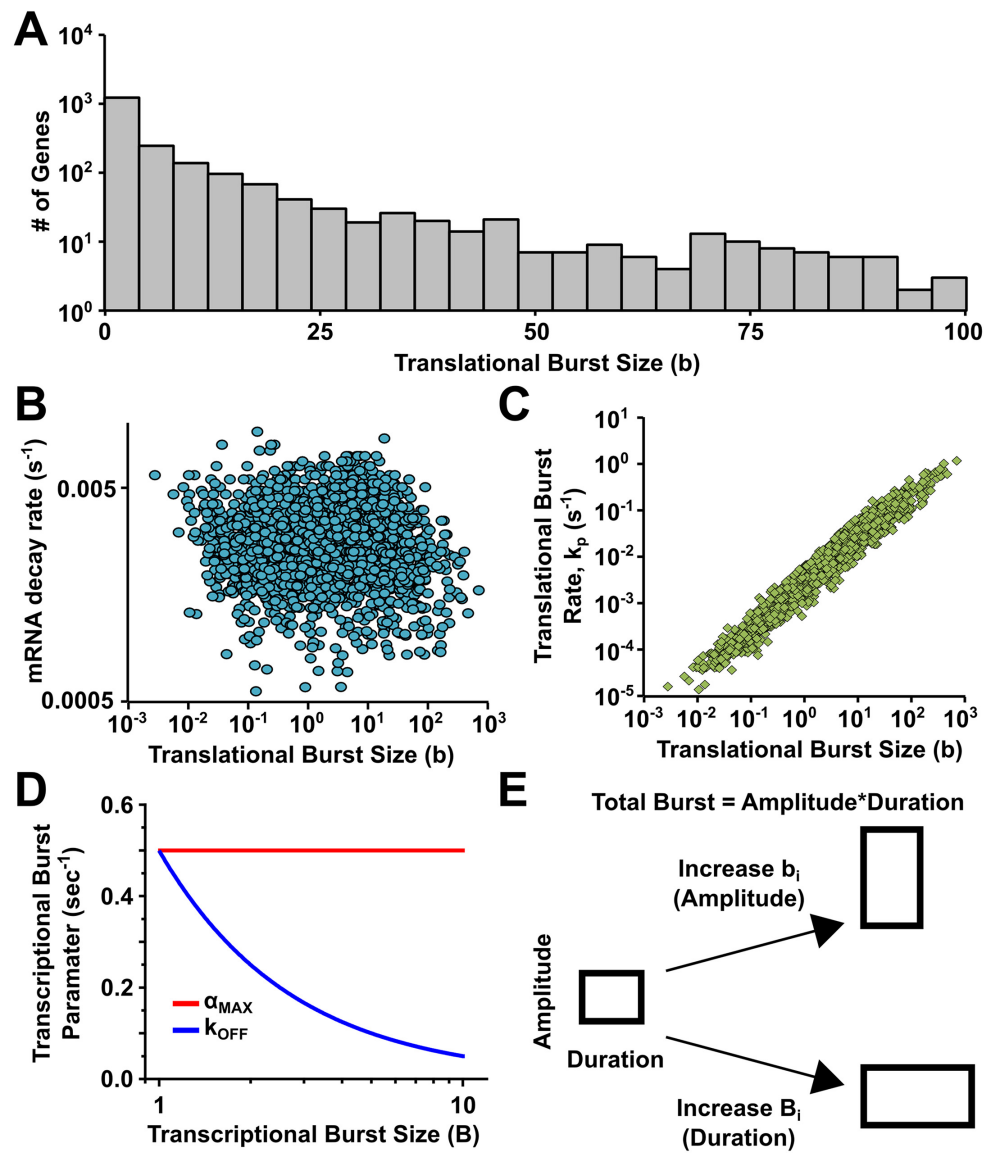
Expression pulse duration is set by transcriptional bursting and pulse intensity is set by translational bursting. **(A)** Histogram of the number of genes with a given translational burst size. **(B)** Plot of the relationship between translational burst size, b, and the mRNA half-life, *γ*_*m*_, for 2077 mRNA in *E*. *coli*. **(C)** Plot of the relationship between translational burst size, b, and the translational burst rate, *k*_*p*_, for 2077 mRNA in *E*. *coli*. **(D)** Plot of the relationship between transcription rate, *α* (red), and the rate of promoters transitioning into the OFF, G0 state, *k*_*OFF*_ (blue), versus the range of calculated B in Fig 3. Here transcription rate was assumed near the maximal physiological limit [15] and k_OFF_ was calculated accordingly. **(E)** Total expression burst is determined by the duration and amplitude of transcription and translation. Transcription predominately sets duration while translation sets amplitude.

Transcriptional and translational bursting are serial processes with a translational burst amplifying the size of a transcriptional burst. To determine the protein specific (indexed by subscript i) translational burst sizes (b_i_) in *E*. *Coli*, reported protein abundances, <P_i_> [35], mRNA abundances, <M_i_> [36], and decay/dilution rates (γ_mi_ and γ_pi_ [36]) were used to estimate values of b_i_ using the relationship (S1 File)

$$b_{i}=\frac{\gamma_{p_{i}}}{\gamma_{m_{i}}}\frac{<P_{i}>}{<M_{i}>},$$ (3)

and we found translational burst sizes that varied over three orders (from ~ 0.1 –~100) of magnitude (Fig 2A). The translational burst size is the product of two parameters–a rate parameter (${\text{k}}_{p_{i}}\;=\gamma_{p_{i}}\frac{<P_{i}>}{<M_{i}>}$, the rate of translation) and a duration parameter $\left(\frac{1}{\gamma_{m_{i}}}\right)$ such that

$$b_{i}\equiv \frac{{\text{k}}_{p_{i}}}{\gamma_{m_{i}}}.$$

In *E*. *coli* the translational burst size is controlled primarily by translation efficiency rather than burst lifetime (Fig 2B and 2C). This finding is in agreement with previous studies showing that translational burst size heavily depends on mRNA structure and sequence [37–39], and variability of translational burst sizes have even been implemented to control protein expression and noise in a precise fashion [40–42]. Thus translational bursting would act to modulate the intensity, and not the duration, of an expression burst.

Similar to translational bursting, transcriptional burst sizes are determined by a rate parameter and a duration parameter:

$$B_{i}\equiv \alpha_{i}T_{ON_{i}}$$

where α_i_ is the rate of transcription during a burst and $T_{ON_{i}}$, inversely proportional to k_OFF_, is the average duration of a burst in the transcribing state (Fig 1B). As previous measurements have shown α_i_ to remain nearly constant over the entire expression range [15], transcriptional burst size is modulated primarily through $T_{ON_{i}}$ (Fig 2D). Therefore, B_i_ is modulated through changes in duration and not intensity. In further support of duration modulation is the finding that the constant value of α_i_ is near the maximum physiological rate of transcription [15], suggesting that during a burst the transcription rate is near the saturation rate. These data establish the orthogonal roles of translation and transcription in bursty expression, whereby translation controls the intensity and transcription controls the duration of expression bursts (Fig 2E).

### Translational burst rate increases with increasing protein abundance and initiates a noise floor

Protein abundance is driven by protein decay/dilution rate (γ_Pi_), the burst size (i.e. b_i_, B_i_, or both) or the frequency (f_B_) of bursts, or

$$<P_{i}>=\frac{b_{i}B_{i}f_{B}}{\gamma_{P_{i}}}$$ (4)

**Fig 3:**
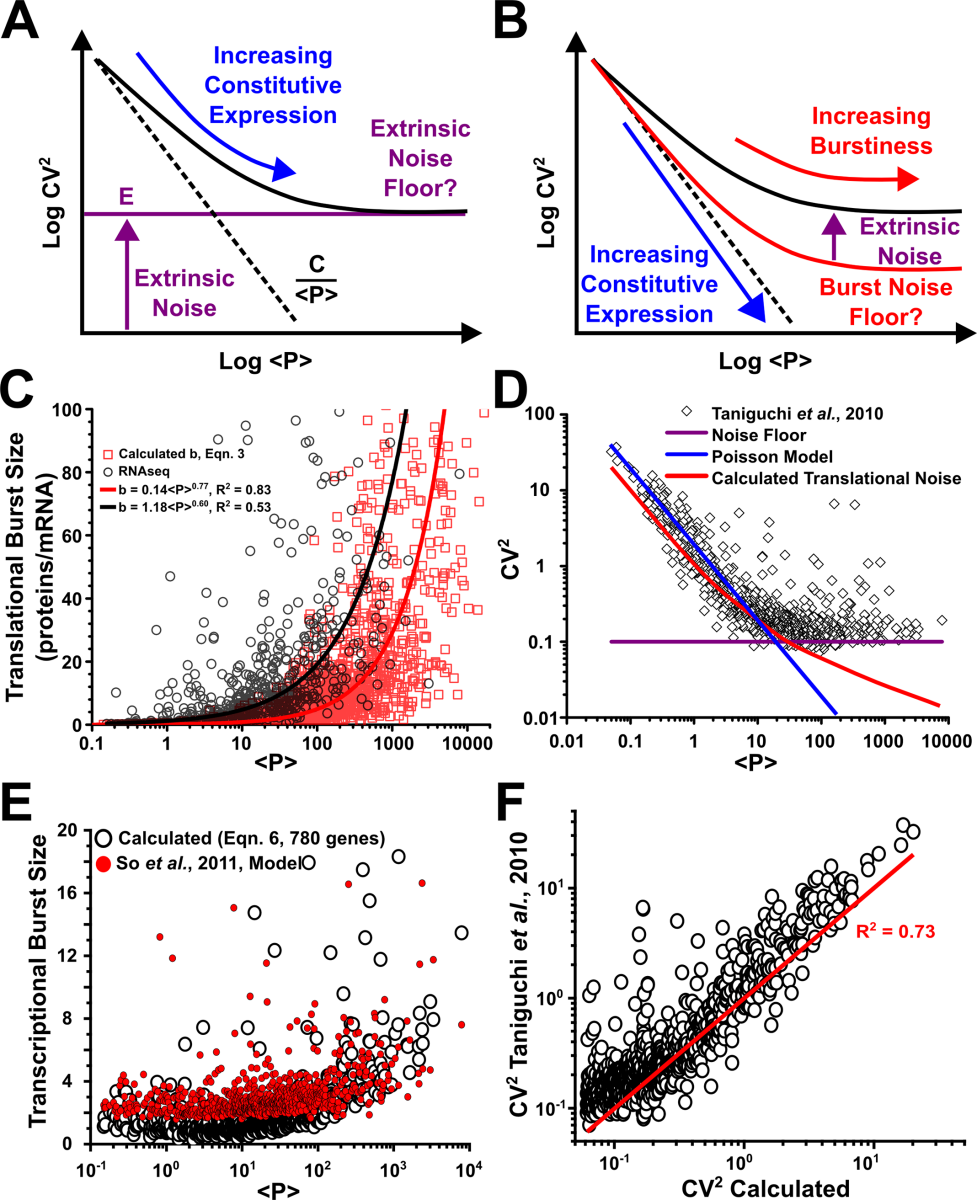
Bursty expression increases with abundance and determines the noise structure observed throughout the *E*. *coli* genome. **(A)** Traditionally, in a plot of CV^2^ versus abundance, <P>, noise in gene-expression is thought to scale as C/<P> (dashed line) and extrinsic noise creates a floor (purple line) with height E. **(B)** Alternatively, the noise floor can be set by increasing burstiness in gene expression for increasing abundance. Extrinsic noise (purple arrow) coupling into bursty expression would increase the level, but not set the noise floor. **(C)** Translational burst sizes versus abundance of the *E*. *coli* proteome (black circles and red squares) fit to power functions. Circles represent the calculated values from Eq 3. Squares represent previously reported RNAseq measurements [4]. **(D)** Plot of CV^2^ and <P> for proteomic *E*. *coli* data (black diamonds, [4]). The calculated translational burst noise (red line) is generated by holding B constant (= 1) and only modulating b. Poisson model (blue line) and noise floor (purple line) are also shown. **(E)** Plot of transcriptional burst size (B_i_) from Eq 6 for 780 genes (open circles) compared to model of measured results from So *et al*. (filled red circles, [15]). **(F)** Plot of measured noise from Taniguchi *et al*. [4], versus the calculated noise (Eq 8) based on fits from **(C)** and **(E)**.

Substituting Eq (4) into Eq (1) yields

$$CV_{i}^{2}=\frac{\gamma_{P_{i}}\left(b_{i}+1\right)}{b_{i}B_{i}f_{B}}\left(B_{i}\right)+E\approx \frac{\gamma_{P_{i}}}{f_{B}}+E,$$

where the approximate relationship holds for *b*_*i*_ >> 1. For *E*. *coli* we may assume that protein decay/dilution is dominated by the cell cycle time and is constant for all proteins, in which case protein abundance is controlled primarily by either burst size or frequency. If increasing abundance is driven by larger burst frequencies (i.e. b_i_ and B_i_ remain constant for all genes), the intrinsic noise would follow the familiar 1/<P> relationship and the appearance of a noise floor would be indicative of a constitutive extrinsic noise (Fig 3A). Conversely, if increasing protein abundances are driven by increases in burst size (i.e. if the burst frequency saturates at a constant value), CV^2^ approaches a constant value (i.e. a floor) with increasing protein abundance. In light of recent experimental studies finding frequency saturation [3, 6], it seems likely that at least a portion of the noise floor is generated by burst noise (Fig 3B). While it has often been assumed that the dominant contribution to the noise floor has come from constitutive extrinsic noise, a careful accounting of the burst noise contribution is required to test this assumption.

While it is clear that there is a large range of translational burst sizes (Fig 2A), the critical question is the relationship between protein abundance and translational burst size. The constitutive extrinsic noise hypothesis rests upon the assumption of translational burst sizes that are invariant with protein abundance. The analyses that have concluded [4] or assumed [3] that noise floors are generated by constitutive extrinsic fluctuations have used this assumption. In contrast, we find a strong correlation (R^2^ > 0.6) between translational burst size and protein abundance in *E*. *coli* (Fig 3C) such that

$$\begin{array}{c} b_{i}=0.126{\langle P_{i}\rangle }^{0.915},\;\langle P_{i}\rangle <10\\ b_{i}=0.202{\langle P_{i}\rangle }^{0.704},\;\langle P_{i}\rangle \;\geq 10. \end{array}$$ (5)

Here a two domain fit was used at <P> = 10. However, this translational burst relationship relies on global measurements of RNA abundance determined using transcriptional shutoff. These measurements vary significantly from laboratory to laboratory, especially those experiments carried out using DNA microarrays in the early days of that technology. To verify this systematic variation in translational efficiency we looked at independent genome-wide measurements by Taniguchi *et al*. [4] for 558 genes for which both the number of proteins produced per mRNA were quantified using RNAseq and protein copy number were measured using calibrated single-cell fluorescence distributions for single molecule copy numbers. A power function fits the results well (R^2^ = 0.53) showing very similar systematic variation in translational efficiency to the database measurements (black versus red solid lines, Fig 3C).

Since translational burst size increases with increasing abundance, we then calculated the translational burst noise (first term on right hand side of Eq 1 with B_i_ set to 1) using b_i_ as given by Eq 5. The translational burst noise was found to account for almost all of the measured noise up to <P_i_> ~ 100, and importantly the initial deviation of CV^2^ from the Poissonian trend is entirely explained by an increasing translational burst rate (Fig 3D). Any contribution to the CV^2^ from either transcriptional bursting or constitutive extrinsic sources must be small for these low and moderate protein populations. *It is important here to note that departure from the Poissonian scaling of noise and the initial flattening of the noise versus abundance curve in E*. *coil is caused by the measured relationship between the translational burst rate and protein abundance and not by constitutive extrinsic noise*. However, at higher protein populations, the measured CV^2^ does diverge from the translational burst noise fit (Fig 3D). The testable theory put forth here is that transcriptional bursting in concert with translational bursting accounts for the continuation of the observed noise floor at the highest expression levels.

### Transcriptional bursting works in concert with translational bursting to maintain the noise floor at highest expression levels

The divergence of the measured CV^2^ from the translational burst noise at higher protein population levels could be due to transcriptional bursting, constitutive extrinsic noise, or a combination of the two. However, recent experiments have demonstrated that transcription of highly expressed genes occurs in stochastic bursts in bacteria [4, 9, 15, 43] and eukaryotic cells [6, 8], and a general mechanism for mediating this transcriptional bursting in *E coli* was recently reported [44]. To explore the source of the noise at the highest expression levels, we calculated the transcriptional burst sizes that would be needed to assign all of the remaining noise to transcriptional bursting. In this case (Fig 3E)

$$B_{i}=<P_{i}>\frac{CV_{i}^{2}}{\left(b_{i}+1\right)}.$$ (6)

These hypothetical transcriptional burst sizes were compared to those predicted by an equation derived from a fit to experimental measurements in *E*. *coli* for 20 different promoters (endogenous and phage) covering a wide range of expression levels [15] (Fig 3E). For moderate values of <P>, the B values predicted by Eq 6 are ~1 and slightly lower than those predicted from the experimental measurements by So *et al*. [15], suggesting that Poissonian expression of mRNA persists for <P> approaching 100. Over the entire <P> range, Eq 6 and the So *et al*. predictions are highly correlated (Figure A in S1 File). At higher levels of <P>, Eq 6 predicts a slightly higher B (Eq 6 predicts a median B of 7 over the highest decade of <P>, while the So *et al*. model predicts a median B of 6, S1 File). This slight difference aside, Eq 6 would seem to be a reasonable estimate of transcriptional bursting in *E*. *coli*, and is well described by (Fig 3E).

$$\begin{array}{c} B_{i}=1\;,\langle P_{i}\rangle <100\\ B_{i}=0.504{<P_{i}>}^{0.368}\;,\langle P_{i}\rangle >100 \end{array}$$ (7)

A careful look at expression bursting shows that the observed CV^2^ floor at high protein populations is at least partially the product of increased expression burstiness, and that by using transcriptional burst sizes consistent with measurements, *the entire noise floor can be attributed to bursty expression*.

To investigate further, we derived an analytical expression for CV^2^ from Eq 1 (using Eqs 5 and 7 for translational and transcriptional bursting) and neglected constitutive extrinsic noise. The noise structure across the genome in *E*. *coli* may then be described by a three-region analytical expression for CV^2^:

Region 1: Poissonian regime 〈*P*_*i*_〉 < 10

$$CV_{i}^{2}\approx \frac{0.126{\langle P_{i}\rangle }^{0.915}\;+1}{<P_{i}>}\approx \frac{1}{<P_{i}>}$$

Region 2: Translational burst regime 10 ≤ 〈*P*_*i*_〉 < 100

$$CV_{i}^{2}\approx \frac{0.202{\langle P_{i}\rangle }^{0.704}\;+1}{<P_{i}>}$$

Region 3: Combined burst regime 〈*P*_*i*_〉 ≥ 100

$$CV_{i}^{2}\approx \left(0.504{<P_{i}>}^{-0.632}\right)\left(0.202{\langle P_{i}\rangle }^{0.704}\;+1\right)$$ (8)

Eq 8 provides a good fit (R^2^ = 0.73) to the measured [4] CV^2^ data (Fig 3F) and indicates at most a minor role for constitutive extrinsic noise in setting the noise floor. *Furthermore*, *our analysis based on the scaling of translational burst size to protein abundance is able to reconcile independently measured noise distributions at the mRNA and protein levels and provides for the first time an unambiguous methodology for inferring transcriptional burst dynamics from protein abundance distribution data*.

### Burst noise models explain measured results better than constitutive extrinsic noise models

**Fig 4:**
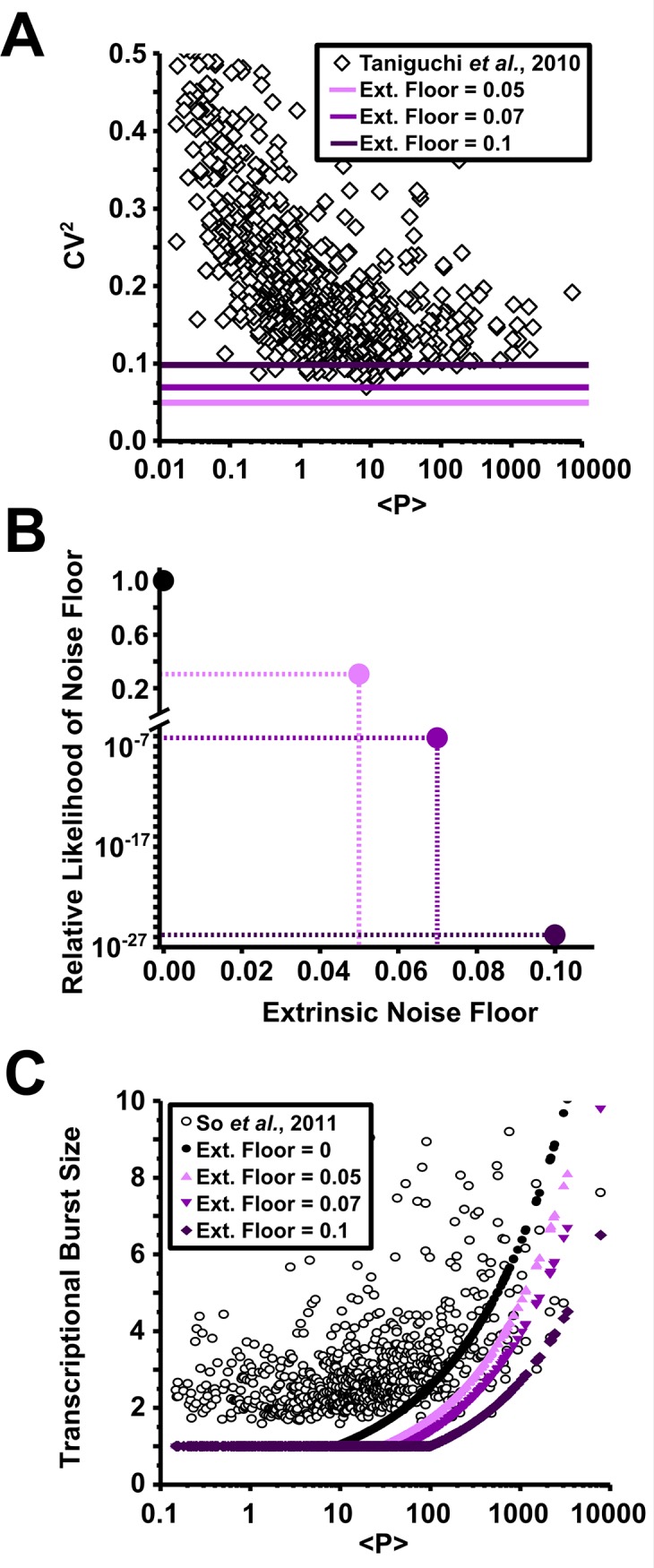
The noise floor is not determined by extrinsic noise acting alone; rather noise from bursty gene expression dominates. **(A)** Illustration of noise floors resulting from various levels of extrinsic noise. **(B)** Relative likelihood of gene expression noise models with various levels of extrinsic noise as evaluated by the Akaike information criteria [45]. The model with extrinsic noise E = 0 has the highest likelihood; models with E = 0.07 and E = 0.1 have extremely low likelihood. **(C)** Transcriptional burst size (B) corresponding to different levels of assumed extrinsic noise. Burst size corresponding to larger noise floors are incompatible with values calculated from the experimentally based model of So *et al*. (2011).

Next, we attempted to create various gene expression models with realistic levels of bursting and non-negligible levels of constitutive extrinsic noise (Fig 4A). Various models were compared according to their ability to represent CV^2^ data with minimal loss of information as evaluated by the Akaike information criteria (S1 File and [45]). Models were fit to the data via power law expressions, similar to Eq 7, relating transcriptional burst size B_i_ to the mean protein level <P_i_>. To make the models as flexible as possible in their ability to accommodate significant levels of extrinsic noise, we allowed complete flexibility in the parameters of the power law function. We found that information loss of the model represented in Eq 1 increases (i.e., the likelihood of the model being accurate decreases) with increasing magnitude of the extrinsic noise floor E (Fig 4B). The presence of a low constitutive extrinsic noise floor of E ≤ 0.05 –a level consistent with partitioning [24] and cell-cycle variation [25] noise–could not be completely ruled out based on the relative likelihood of this model (0.3) compared to a noise floor of zero; however, models with levels of extrinsic noise close to the observed noise floor (E = 0.07 and 0.10) could be conclusively excluded based on excessive information loss. The effect of assuming an increased constitutive extrinsic noise was to decrease calculated transcriptional burst sizes (Fig 3E) to levels that were inconsistent with those observed in So *et al*. [15] and Taniguchi *et al*. [4]. Most strikingly, similar results were obtained from evaluation of various values of E using the full two-state model (i.e. without the simplifying assumptions in Eq 1; S1 File), demonstrating that these conclusions are not dependent on either model of gene expression. This analysis indicates that a burst-driven noise floor (region 3 in Eq 8) theory is a much more likely explanation for the observed noise behavior than the accepted constitutive noise floor model.

### Noise in other organisms is more targeted than in *E*. *coli*

**Fig 5:**
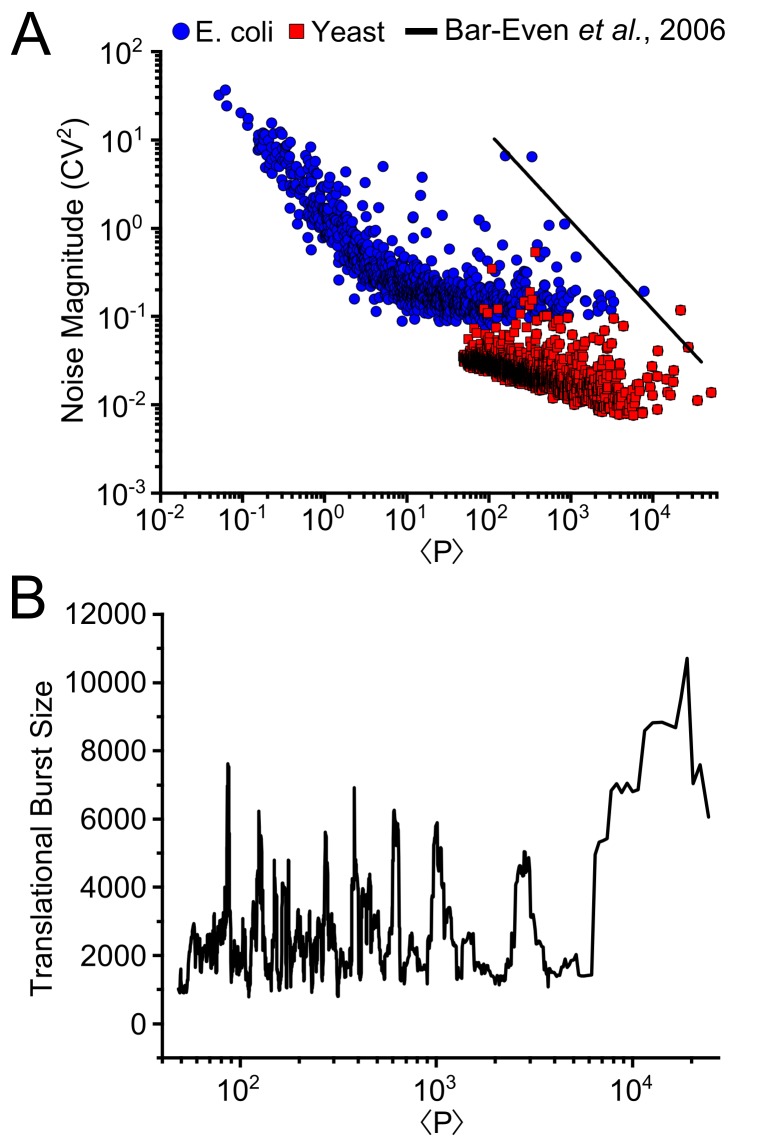
Yeast shows less burstiness and no noise floor compared to *E*. *coli*. **(A)** Reported noise magnitude measurements for 1467 genes of *S*. *cerevisiae* plotted along with genome-wide *E*. *coli* noise measurements from Fig 3D. **(B)** Using calculated values for translational burst size [1] based off of four separate databases [47–50], in contrast to *E*. *coli*, the translational burst size are invariant to protein abundance. A moving average of 20 genes was applied to the trend.

The idea of a burst-driven floor is intriguing in *E*. *coli*, and since bursty expression has been observed across many domains of life, we hypothesized that the noise structure in other organisms may also be dominated by bursty expression. To check for this, we reanalyzed the noise behavior of *Saccharomyces cerevisiae* since noise has been measured for more than 1,000 different proteins in the high abundance regime where a constitutive extrinsic noise floor would be found [32, 33]. Despite differences in reported noise level for a given protein abundance, both studies demonstrate that the noise in *S*. *cerevisiae* continues to decline with increasing protein abundance to levels significantly lower than found in *E*. *coli* (Fig 5A) [32, 33]. The contrast in noise structure likely arises from the non-systematic variation in translational burst size in *S*. *cerevisiae* over most of the protein abundance range (Fig 5B), unlike what is found in *E*. *coli* (Fig 3C). The constitutive extrinsic noise floor for *S*. *cerevisiae* was found to be approximately 0.01 using a two-reporter technique [32, 46], a level that is consistent with our finding of constitutive extrinsic noise ≤ 0.05 in *E*. *coli* and likelihood analysis (Fig 4). It appears that *E*. *coli* and *S*. *cerevisiae* achieve high levels of expression through different mechanisms. In *E*. *coli* high expression levels are achieved by increases in translational burst size at a saturated transcriptional burst frequency, while in *S*. *cerevisiae* translational burst size remains fairly constant while transcriptional burst frequency continues to increase. However, high noise–at a level around the more uniform noise floor in *E*. *coli*–is found for a select group of proteins in *S*. *cerevisiae* (Fig 5A). So for *S*. *cerevisiae* high noise at high abundance is promoter-specific [30, 34] and is most often found associated with stress response [33].

**Fig 6:**
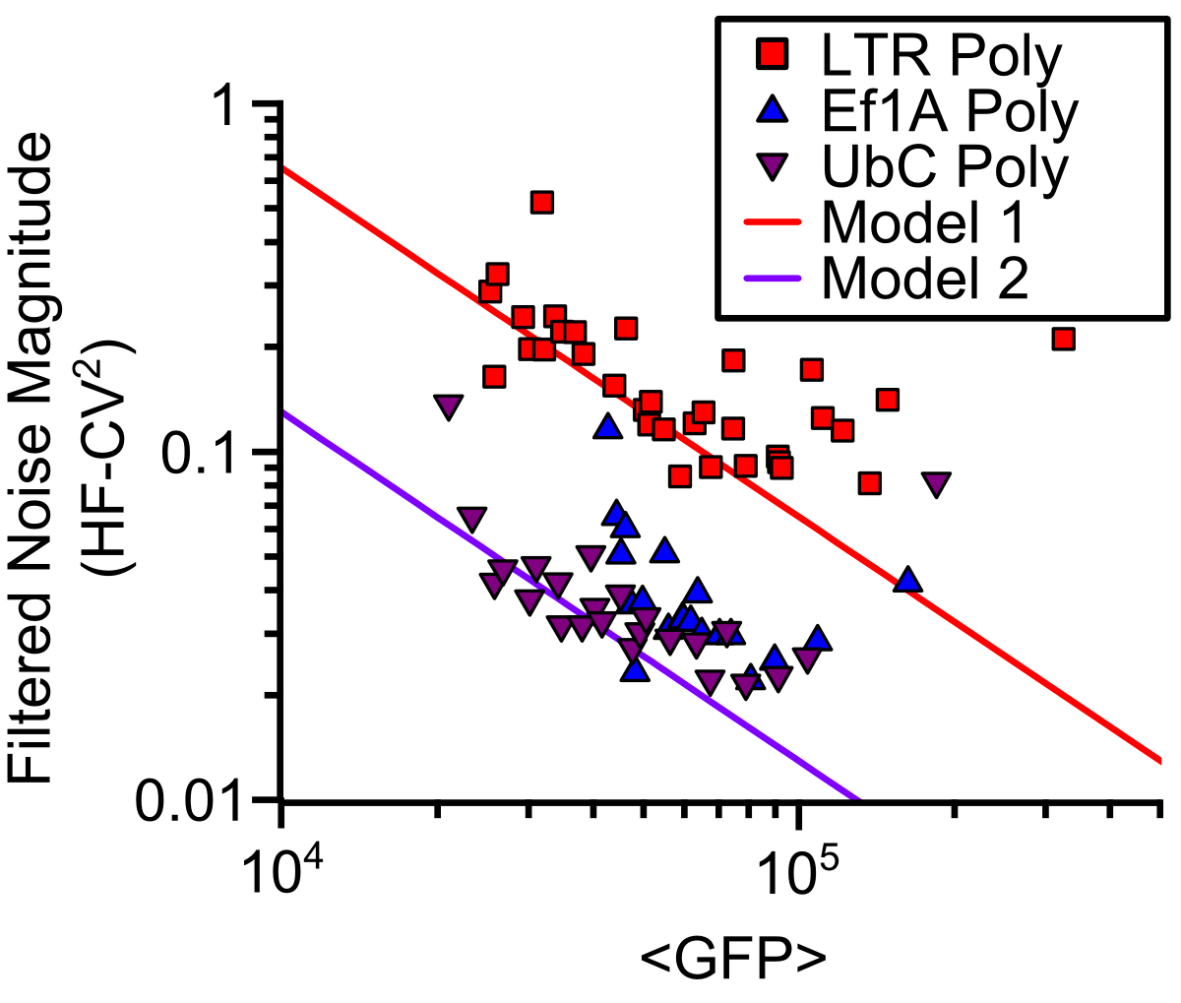
Evidence of the noise floor at high abundance in mammalian cells. Polyclonal populations of T cells infected with a viral HIV-LTR and housekeeping promoters, UbC and Ef1A, show an increase of noise at higher abundances. Time-lapse microscopy and signal processing of limited duration experiments filters extrinsic noise (High-frequency or HF-CV^2^, [6]) suggesting that burstiness drives the noise increase from a simple model line that is inversely proportional to mean GFP. Data adapted from Dar *et al*., 2012, [6].

To explore the promoter-specific role in distributing noise, we used recently reported data utilizing a method for measuring the noise behavior of individual promoters across thousands of integration sites in human T cells [6]. This method allows measurement of expression noise of the same promoter at many different expression levels (i.e. in different chromosomal integration sites) while keeping most genetic circuit parameters (e.g. mRNA and protein lifetimes; translational burst rate) constant. Data from Dar *et al*., 2012 included the noise behavior of the HIV long terminal repeat (LTR) promoter–which is known to exhibit significant transcriptional bursting [6, 20]–and two housekeeping promoters (Ef1A and UbC) (Fig 6). Both the bursty LTR promoter and the more constitutive (less noisy) promoters appear to approach noise floors at high expression levels, but much like the contrast between *E*. *coli* and *S*. *cerevisiae*, these floors are separated by about an order of magnitude. Showing similarity to *E*. *coli*, burst size of the LTR across diverse integration sites is dominated by changes in promoter activity duration (k_OFF_) and not level (α) (Fig 2D and Figure B in S1 File). Importantly, the LTR, Ef1A, and UbC noise behaviors reported here (Fig 6) are not likely related to constitutive extrinsic fluctuations. Time-lapse fluorescent microscopy was used to measure both the magnitude and the dynamics (i.e. frequency content) of the expression noise. Extrinsic noise is known to reside in a lower frequency regime than the intrinsic noise in *E*. *coli* [51–53] which is believed to be due to the additional low-pass filtering extrinsic noise experiences as it is processed first through its own molecular network and then subsequently through the intrinsic gene circuit. By analyzing only the higher frequency noise components [6], it is likely that the noise floors shown here can be ascribed to intrinsic fluctuations since the same double filtering of extrinsic noise takes place in mammalian cells.

## Discussion

The analysis in Figs 3 and 4 demonstrates that in *E*. *coli* the expression noise is dominated by translational and transcriptional burst noise, and the noise floor–CV^2^ approaching a constant value at high expression levels–is primarily set by bursting (Fig 3B). Furthermore, although transcriptional bursting appears to be the focus of much contemporary research, it actually plays a fairly minor role in the genome-wide noise behavior. Instead, *translational efficiency (i*.*e*. *translational burst size) is the more potent force and the measured relationship between the translational burst size and protein abundance described here is enough–even in the absence of transcriptional bursting–to negate the hypothesis of a substantial constitutive extrinsic noise floor*. Instead of global fluctuations, the noise floor is indicative of burst frequency saturation and the direct coupling between protein abundance and burst size (Figs 3 and 4). This abundance-burst size coupling appears to be a uniform constraint that sets a global noise limitation on *E*. *coli*.

In contrast, the noise structure in *S*. *cerevisiae* is much less uniform. Instead noise for many proteins continues to decline, ultimately approaching a very low constitutive extrinsic noise floor. On the surface these results would seem to say that *E*. *coli* is burstier (i.e. has bigger expression bursts) than *S*. *cerevisiae*. However that idea may be quickly dismissed by noting that translational burst sizes in *S*. *cerevisiae* (Fig 5B), even for low abundance proteins, is larger than the combined (translational and transcriptional) burst sizes in *E*. *coli*. Instead, it seems that transcriptional burst frequency has not saturated in *S*. *cerevisiae*. Longer mRNA and protein lifetimes and perhaps longer duration of expression bursts in *S*. *cerevisiae* may also contribute to a smaller burst noise effect.

The T cell results show an interesting mix of behaviors that mimic some aspects of both *E*. *coli* and *S*. *cerevisiae*. Like *E*. *coli* all three promoters studied in T cells exhibited expression noise that approached noise floors that were not related to constitutive extrinsic noise (Fig 6). These floors are indicative of burst frequency saturation and of a switch from an increasing burst frequency to an increasing burst size (Figure B in S1 File and [6]). Yet like *S*. *cerevisiae* the magnitudes of these noise floors are promoter-specific with a bursty promoter (LTR) having a significantly higher noise floor than more constitutive promoters (UbC, Ef1A). This implies that different promoters saturate at different transcriptional burst frequencies, and that promoters with larger burst sizes may saturate at lower burst frequencies than promoters with smaller burst sizes.

The significant fallout from these results is the elimination of many inconsistencies that have muddied the analytical framework that connects transcriptional processes and the noise observed in the protein populations. Inferential methods did not fail because of any inherent shortcomings in the two-state model, but instead suffered from inaccurate assumptions about constitutive extrinsic noise and translational bursting. Correcting these assumptions leads to consistent results, for example showing agreement between burst sizes measured at the mRNA level and those inferred from protein noise measurements (Fig 1C).

The results presented here demonstrate that noise floors are indicative of burst frequency saturation, and not fluctuations in global resources, raising an intriguing question: is frequency saturation and the resultant noise floor a constraint (i.e. the unavoidable consequence of global gene expression in a shared resource environment) or can they be independently manipulated? This question cannot be explored by the manipulation of the fluctuations of individual genes [54, 55], but would instead require ways to manipulate the global structure of noise, and more specifically would require manipulation of the maximum burst frequency for groups of genes. To explore this question we propose to utilize advances in the bottom-up construction of synthetic systems that mimic cellular attributes of confinement (i.e. size), macromolecular crowding, and expression resource limitations [56].

Recent investigations have reported cell-free expression systems confined in lipid vesicles [57], porous media [58], and microfluidic structures [59]. Although transcriptional bursting has not been the focus of any of these studies, one recent investigation [60] reported the measurement of noise in cell-free expression confined within 20 fL polydimethylsiloxane (PDMS) containers, and demonstrated key technological steps (reproducible fabrication of containment vessels, robust sealing of vessels, and time-lapse fluorescent microscopy over extended periods) that might enable the study of transcriptional bursting using noise analysis methods recently applied to cellular systems [3, 6, 15]. In an additional study, a two-reporter method for quantifying correlated noise [23] was used to characterize stochasticity in gene expression in cell-sized vesicles, and found that measured fluctuations were comparable to levels in *E*. *coli* and mostly an intrinsic property that can be produced in a minimal cell-free system [61]. Importantly, the author’s findings are consistent with the main conclusion of this study, and suggest a strategy for investigating if a noise floor is a constraint or a feature. In such cell-free constructions it would be possible to study noise behavior in systems where known sources of transcriptional bursting such as DNA supercoiling mechanisms and moribund RNAP-promoter complexes [44, 62] can be precisely controlled, eliminated or greatly reduced. If it is true that constitutive (i.e. non-bursty) expression can be achieved in cell-like confined and crowded environments, it should be possible in these synthetic constructs. Conversely, if transcriptional bursting and noise floors prevail even under such favorable conditions, it seems likely that these behaviors arise from fundamental constraints of relatively complex molecular interactions in confined, crowded, and resource limited environments.

There are studies that have decoupled mean abundance changes from noise modulation in individual genes to elucidate the advantageous role of noise in organism fitness [63–65]. However, a remaining question is if similar advantages accrue at the global scale, i.e. might it be advantageous for an organism to distribute high noise across a variety of genes? *E*. *coli* and *S*. *cerevisiae* illustrate contrasting noise distributions that might be thought of as non-specific (high noise indiscriminately distributed to all high abundance proteins, i.e. *E*. *coli*) and specific (high noise distributed to a select group of high abundance proteins, i.e. *S*. *cerevisiae*). This select group of noisy proteins in *S*. *cerevisiae* has been strongly associated with gene-specific promoter and regulatory arrangements that couple together responsiveness and noise [66, 67] and stress responses [33]. The distribution of noise to stress response genes may suggest a bet-hedging strategy where populations mitigate environmental fluctuations through a noise-mediated assignment of some cells into alternate phenotypes. At the single gene scale it has been shown experimentally that frequency matching between environmental and gene expression fluctuations provides a fitness advantage to populations [65]. This frequency matching behavior is especially intriguing in light of the finding here that noise floors are indicative of burst frequency saturation. We hypothesize that the burst frequency saturation level and the resultant noise floor may adapt to fluctuating environments, and we propose that adaptation experiments would be well suited for elucidating if global noise structure may be a conserved feature. By placing organisms in environments that stress multiple pathways (or ideally affect the cell on a global scale), adaptation of the noise floor to different stress levels and fluctuation frequencies would imply that global noise structure is a feature capable of improving fitness. Such global noise modulating experiments will define the role of the conserved burst noise structure across organisms and address whether modulating noise above and below the floor has functional consequences.

## Materials and Methods

### Calculations

Eq 1 was derived assuming that k_OFF_ >> k_ON_, thereby allowing each expression burst (transcription and translation taken together) to be approximated as the product of 3 uncorrelated random processes: Process A composed of a Poissonian pulse train of impulse functions of weight = 1 having an average value $\bar{A}$ (transcriptional initiation, i.e. burst frequency); Process B (transcriptional bursting) with a mean value of $\bar{B},$ and a variance of $\sigma_{B}^{2};$ and Process b (translational bursting) with a mean value of $\bar{b},$ and a variance of $\sigma_{b}^{2}$. The Fano factor (FF) of this composite process–and therefore the FF expected in the protein population (FF_<P>_)–is (S1 File):

$$FF_{<P>}\;=\;\left(\bar{b}+FF_{b}\right)\left(\bar{B}+FF_{B}\right),$$

where *FF*_*b*_ is the Fano factor of the translational burst size and *FF*_*B*_ is the Fano factor of the transcriptional burst size. In the absence of constitutive extrinsic noise, *FF*_*b*_ = 1, the value of *FF*_*B*_ is model dependent and may vary between ~0 (for small B) and 1 (S1 File). Eq (1) uses the model that allows for a smooth transition from Poissonian expression (i.e. constitutive transcription with no bursting) to bursty expression (S1 File). In this model, Poissonian expression is simply $\bar{B}=1$ and *FF*_*B*_ = 0. Accordingly, Eq 1 uses the relationship (neglecting constitutive extrinsic noise)

$$FF_{<P>}\;=\;\bar{B}\left(\bar{b}+1\right).$$

The expression for translational burst rate was derived using the steady state equation for mean protein abundance <P> = (α*k_p_)/(γ_m_*γ_p_), where α and k_p_ are the transcription and translation rates respectively, and γ_m_ and γ_p_ are the mRNA and protein degradation rates respectively. Upon rearrangement and substitution of b = k_p_/γ_m_ and <M> = α/γ_m_, Eq 3 is reached. Here the protein decay in *E*. *coli* was assumed to be dominated by dilution caused by cell growth. A constant cell doubling time of 55 minutes was used.

For comparing calculated burst size values of Eq 6, an equation fit to experimental measurements on 20 *E*. *coli* promoters measured by So *et al*., [15] was used: B = 1 + 1.5*<M>^0.64^. The same literature values for <M_i_> used to calculate b_i_ were used in this calculation [36]. Burst size values were then plotted against their database <P_i_> values for the comparison shown in Fig 3D.

## Supporting Information

S1 File Supporting Information PDF file. (PDF)

S1 Table Supplementary Data Spreadsheet. (XLSX)
