# Supplementary material for: The Low Noise Limit in Gene Expression
Source: PLoS One. 2015 Oct 21;10(10):e0140969. doi: 10.1371/journal.pone.0140969 (PMC4619080; doi:10.1371/journal.pone.0140969)
Supplement: S1 File — (PDF) [file pone.0140969.s001.pdf]

Supporting Information for:

The low noise limit in gene expression

Roy D. Dar, Brandon S. Razooky, Leor S. Weinberger, Chris D. Cox, and Michael L. Simpson*

*Address for correspondence: simpsonML1@ornl.gov

Table of Contents

Supporting Figures 3

Figure A: Comparison of burst size predictions between different models 3

Figure B: Transcriptional bursting in mammalian cells 4

Extended Experimental Procedures 5

Distinguishing between an extrinsic and burst noise floor 5

Forcing an extrinsic noise floor 6

Transcriptional bursting in mammalian cells 8

Expression burst analysis 9

Supplemental References 12

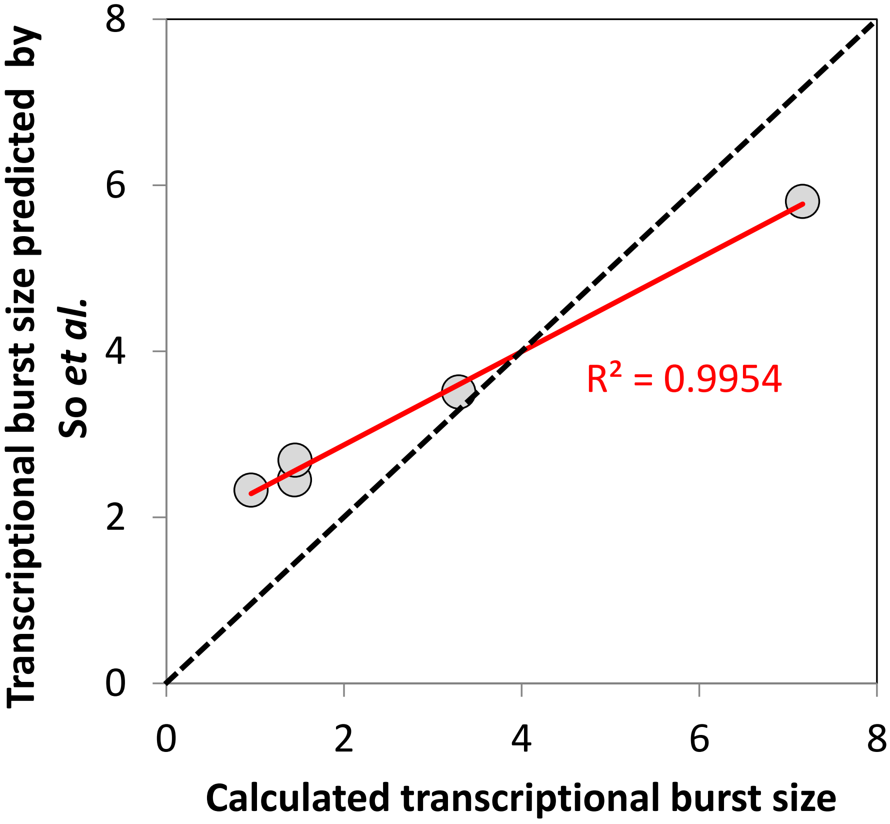

Figure A related to Fig 3. Comparison of burst size predictions between different models. Based on experimental measurements, So *et al.* (So et al., 2011) predict that transcriptional burst size varies with mRNA population (<M_i>) such that

$$B_i \approx 1 + 1.5\langle M_i \rangle^{0.64}.$$

Using the literature values for <M_i> we generated B_i values for *E. coli* using this expression and associated these with their corresponding <P_i> values (see the Supplemental Spreadsheet).

In Fig. 3E of the main text we compare our predicted values of B_i – derived directly from the measured CV^2 data – with those predicted by the So *et al.* equation as a function of protein abundance. In the figure above the So *et al.* values are plotted versus our predicted values. The B values in this graph are the median values taken over decades of protein abundance (i.e. the lowest B values are the median values found in the protein population from 0.1 to 1.0; the highest points are for the protein population from 1000 to 10,000). The black dashed line in this graph represents the x=y line. Our predicted values are highly correlated with the So *et al.* predictions (R^2 = 0.995).

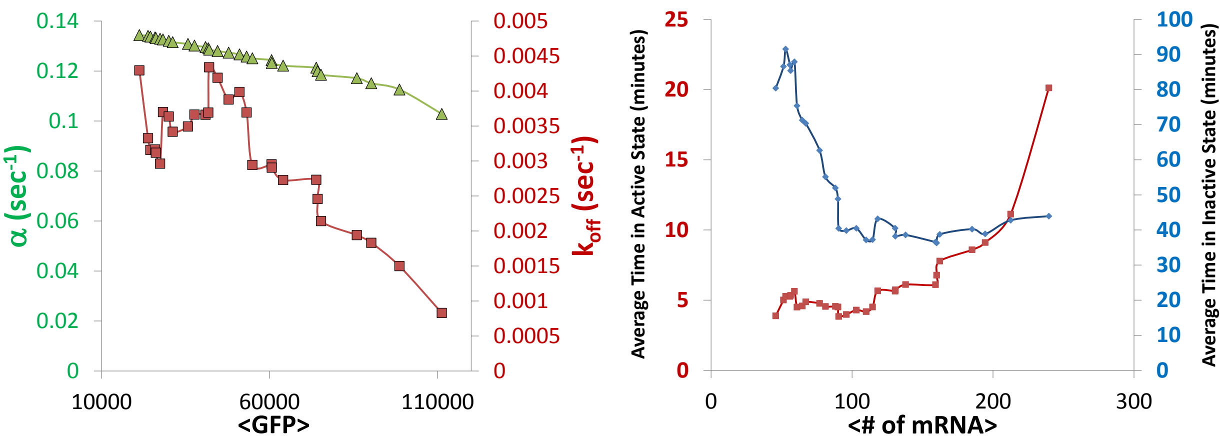

Figure B related to Fig 6. Transcriptional bursting in mammalian cells. For genome-wide transcription of the HIV-LTR promoter, burst size is dominated by changes in k_{OFF} and not transcription rate. (left) The k_{OFF} trend decreases by a factor of 4 while the α trend only slightly decreases. (right) As reported in (Dar et al., 2012), the burst frequency plateaus (average time in the off state approaches a constant value) with increasing mRNA abundance. The burst size increases through increases of the duration of time in the active promoter state.

Distinguishing between an extrinsic and burst noise floor

Eqns. 2 and 4 in the main text show that

$$CV_i^2 = \frac{b_i + 1}{< P_i >}(B_i) + E = \frac{C_1}{< P_i >} + C_2,$$

where C_2 represents a noise floor. Since

$$\langle P_i \rangle = \frac{B_i b_i f_{B,}}{\gamma_p}$$

where f_B is the frequency of transcriptional bursts and γ_p is the protein decay/dilution rate,

$$\frac{\gamma_p (b_i + 1)}{B_i b_i f_B}(B_i) + E \approx \frac{\gamma_p}{f_B} + E.$$

If at the larger values of <P_i> increasing protein abundance is primarily driven by increasing values of b_i and B_i, and γ_p is controlled by a constant cell growth rate, then the transcriptional burst frequency must remain relatively constant, and

$$C_B + E = C_2, \qquad \text{(S-1)}$$

where C_B is a constant value (the ratio of the protein decay rate to the maximum transcriptional burst frequency) that defines the burst noise floor. Eqn. S-1 describes the noise floor as the combination of burst and constitutive extrinsic noise floors. If C_B is large enough, the constitutive extrinsic noise floor must be small.

Forcing an extrinsic noise floor

We tested various models of gene expression noise with significant levels of constitutive extrinsic noise to determine if they could parsimoniously represent the Taniguchi *et al.* (Taniguchi et al., 2010) noise data and transcriptional bursts described by the experimentally based model of So *et al.* (So et al., 2011). We tested the following models:

Model 1 (Equation 1 from the main text):

$$CV^2 = \frac{B(1+b)}{\langle P \rangle} + E$$

Model 2 (two-state model):

$$CV^2 = \frac{1+b+Bb}{\langle P \rangle} + E$$

We obtained values of b from Eqn. 5 in the main text to apply to each of the two models. Average values of B were assumed to be related to protein expression through a power law of the form:

$$B_i = \max(B_{min},\ q\langle P_i \rangle^r)$$

where B_{min} = 1 for Model 1 and B_{min} = 0 for Model 2. Values of q and r were adjusted to obtain a maximum likelihood fit of each model to the noise data of Taniguchi *et al.* (Taniguchi et al., 2010). Log transformations of each model were used to obtain residuals that were near-normally distributed and with magnitude independent of <P>.

Both models were evaluated for values of the extrinsic noise floor of E ranging from 0 to 0.1. To assess the ability of each model to describe the data of Taniguchi *et al.* (Taniguchi et al., 2010) , we used the Akaike information criteria (Akaike, 1974) (AIC):

$$AIC = 2k - 2ln(\mathcal{L})$$

where k is the number of parameters in the model and $\mathcal{L}$ is the likelihood of the model given the observed data. The AIC characterizes the information that is lost when a model is used to represent the underlying process that generates the data. The probability that a given model *j* has minimized the information loss compared to the model with AIC_{min} is given by (Akaike, 1974):

$$\exp\left(\frac{AIC_{min} - AIC_j}{2}\right)$$

and can be considered as a relative comparison of model quality. The log-likelihood $\ln[\mathcal{L}(p, q|r_i)]$ of each model was determined according to:

$$\ln[\mathcal{L}(p,q|r_i)] = \sum_i \ln\left(\frac{1}{\sqrt{2\pi\sigma^2}} e^{-\frac{r_i^2}{2\sigma^2}}\right)$$

where r_i are the residuals from the fit of the log-transformed model to the data of Taniguchi *et al.* (Taniguchi et al., 2010), σ^2 is the variance of the residuals, and the mean of the residuals is assumed to be zero.

Maximum likelihood estimates of the model parameters q and r for each model are summarized below. Power law parameters from Eqn. 7 in the main text and from the power law function determined by So *et al.* (So et al., 2011) are provided for reference.

**Model**	**Maximum Likelihood values of (q, r)**			
	**E=0**	**E=0.05**	**E=0.07**	**E=0.1**
**Model 1**	0.426, 0.389	0.221, 0.444	0.182, 0.444	0.141, 0.427
**Model 2**	0.111, 0.583	0.017, 0.785	–	0.00, 0.00
**Power law from Equation 7 in the main text.**	0.504, 0.368	–	–	–
**Power law from So et al., (2011)**	1.5, 0.64	–	–	–

Results from analysis of Model 1 are presented in the Fig 4 of the main text. For Model 2, the relative likelihood of models with various levels of extrinsic noise are:

	**E=0**	**E=0.05**	**E=0.1**
**Relative likelihood of Model 2 with various levels of extrinsic noise, E.**	1	2.57E-5	5.5E-29

For Model 2, even moderate levels of constitutive extrinsic noise (E=0.05) result in unlikely models. For the highest level of extrinsic noise (E=0.1), the optimum fit was obtained for q=0 and r=0, corresponding to strictly Poissonian mRNA expression across all expression levels and contrary to known transcriptional behavior (So et al., 2011). Therefore, our conclusion that bursty expression plays a major role in establishing the observed noise floor and that the noise floor cannot be the result of extrinsic noise acting alone, does not depend upon a particular model of gene expression noise (Fig 4).

Transcriptional bursting in mammalian cells

Using a high-throughput time-lapse imaging, we previously measured transcriptional burst size and frequency for over 2000 integration sites of a polyclonal population of human T-cells harboring diverse integrations of a single HIV-LTR promoter driving a de-stabilized d2GFP reporter with a 2.5 hour half-life (Dar et al., 2012). Using this data and the reported equations for transcriptional burst size and burst frequency, Bs, BF, and k_{off} are calculated for the HIV LTR-d2GFP polyclonal sub-clusters or groups of single-cell with unique integration sites and similar mean expression levels (Dar et al., 2012). k_{off} is calculated using an assumed low "on fraction" range of $O < 0.2$ and in addition the reported mRNA FISH measurement of 110 mRNA is assumed to be equivalent to $O = 0.1$ and used as a benchmark to calculate the O and k_{off} values (using BF or k_{on}) for each polyclonal sub-cluster by scaling by their <GFP>. Finally $\alpha = Bs*k_{off}$ was calculated and a 5 sub-cluster moving average across abundance levels was applied before plotting the results (Figure B and Fig 6).

Expression burst analysis

If an expression burst (combined transcriptional and translational) occurs in a relatively short time period (i.e. if we consider $k_{OFF} >> k_{ON}$), then we can approximate this as the product of three random processes: Process A (transcriptional initiation) composed of a Poissonian pulse train of impulse functions of weight = 1 and average value $\bar{A}$; Process B (transcriptional bursting) that is uncorrelated with process A, has a mean value of $\bar{B}$, and a variance of σ_B^2; and Process b (translational bursting) that is uncorrelated with processes A and B, has a mean value of $\bar{b}$, and a variance of σ_b^2. The autocorrelation functions of these three processes are

$$\phi_A(\tau) = \bar{A}\delta(\tau) + \bar{A}^2$$

$$\phi_B(\tau) = \sigma_B^2\delta(\tau) + \bar{B}^2$$

$$\phi_b(\tau) = \sigma_b^2\delta(\tau) + \bar{b}^2$$

The autocorrelation function of the expression burst is given by the product of the autocorrelation functions of these three functions or

$$\phi_{ABb}(\tau) = \phi_A(\tau) * \phi_B(\tau) * \phi_B(\tau) = \bar{A}\sigma_b^2\sigma_B^2\delta(\tau) + \bar{A}\bar{B}^2\sigma_b^2\delta(\tau) + \bar{A}\bar{b}^2\sigma_B^2\delta(\tau) + \bar{A}\,\bar{b}^2\bar{B}^2\delta(\tau)$$

where we have neglected all the $\bar{A}^2$ terms because $\bar{A} \ll 1$. From this we get

$$\sigma_{AbB}^2 = \bar{A}\sigma_b^2\sigma_B^2 + \bar{A}\bar{B}^2\sigma_b^2 + \bar{A}\bar{b}^2\sigma_B^2 + \bar{A}\,\bar{b}^2\bar{B}^2$$

and the Fano factor (which would be the Fano factor of the protein abundance) is

$$FF_{AbB} = FF_{<P>} = \frac{\sigma_{AbB}^2}{\bar{A}\,\bar{b}\bar{B}} = \bar{b}\bar{B} + \frac{\sigma_b^2\sigma_B^2}{\bar{b}\bar{B}} + \bar{B}\frac{\sigma_b^2}{\bar{b}} + \bar{b}\frac{\sigma_B^2}{\bar{B}}$$

or

$$FF_{<P>} = \bar{b}\bar{B} + FF_b * FF_B + \bar{B} * FF_b + \bar{b} * FF_B = (\bar{B} + FF_B)(\bar{b} + FF_b),$$

where FF_b and FF_B are the Fano factors of translational and transcriptional burst sizes respectively.

In the absence of constitutive extrinsic noise FF_b=1 and for the two-state model of transcriptional bursting $FF_B = 1$ (Kepler and Elston, 2001; Simpson et al., 2004), so that

$$FF_{<P>} = \bar{b}\bar{B} + 1 + \bar{B} + \bar{b} = (\bar{b} + 1)(\bar{B} + 1).$$

This equation points out that in the two-state model a transcriptional burst size, B = 1 produces a different Fano factor (FF = 2 (1 for the value of $\bar{B}$ and an additional + 1 for the Fano factor of B)) than Poissonian expression of single mRNA molecules.

To overcome this apparent discrepancy in the Fano factor in the two-state model, we introduce a model in which the first mRNA synthesis event begins the burst, and the number of synthesis events that follow the initiating event (B_E) is a random variable. In that case,

$$B = 1 + B_E$$

where the 1 term stems from the Poissonian process of initiation events and B_E is the randomized process contributing to the variance in the burst size. Therefore to recover B, B_E must equal B – *1*, and the variance in B exclusively comes from B_E

$$\sigma_B^2 = \sigma_{B_E}^2 = B_E$$

From this it follows that,

$$FF_B = \frac{\sigma_{B_E}^2}{B} = 1 - \frac{1}{\bar{B}},$$

And at the low end of expression where $\bar{B} \approx 1$,

$$FF_{<P>} \approx \bar{B}(\bar{b} + 1). \quad \text{(S-2)}$$

In contrast to the two-state model, this model provides a smooth transition from Poissonian expression of single mRNA molecules to bursts of multiple mRNA production.

Note that the model presented here is based on a burst of protein expression where the average size of the burst is b*B and the frequency of the burst is driven by the random process A as described above. These conditions can be violated when b << 1, where almost regardless of the value of B, protein expression is nearly Poissonian. In such cases – which all occur at low values of <P> – CV^2 goes as 1/<P> (the Poissonian regime in Eqn. 8 of the main text). Since noise

behavior is so insensitive to transcriptional burst size in this regime, it is difficult to extract accurate values of B from the protein noise for the lowest protein populations.

REFERENCES

Akaike, H. (1974). A new look at the statistical model identification. Automatic Control, IEEE Transactions on *19*, 716-723.

Dar, R.D., Razooky, B.S., Singh, A., Trimeloni, T.V., McCollum, J.M., Cox, C.D., Simpson, M.L., and Weinberger, L.S. (2012). Transcriptional burst frequency and burst size are equally modulated across the human genome. Proc Natl Acad Sci U S A *109*, 17454-17459.

Kepler, T.B., and Elston, T.C. (2001). Stochasticity in transcriptional regulation: Origins, consequences, and mathematical representations. Biophys J *81*, 3116-3136.

Simpson, M.L., Cox, C.D., and Sayler, G.S. (2004). Frequency domain chemical Langevin analysis of stochasticity in gene transcriptional regulation. Journal of theoretical biology *229*, 383-394.

So, L.H., Ghosh, A., Zong, C., Sepulveda, L.A., Segev, R., and Golding, I. (2011). General properties of transcriptional time series in Escherichia coli. Nature genetics *43*, 554-560.

Taniguchi, Y., Choi, P.J., Li, G.W., Chen, H.Y., Babu, M., Hearn, J., Emili, A., and Xie, X.S. (2010). Quantifying E-coli Proteome and Transcriptome with Single-Molecule Sensitivity in Single Cells. Science *329*, 533-538.
